# Clarification of electrical current importance in plasma gene transfection by equivalent circuit analysis

**DOI:** 10.1371/journal.pone.0245654

**Published:** 2021-01-28

**Authors:** Yugo Kido, Hideki Motomura, Yoshihisa Ikeda, Susumu Satoh, Masafumi Jinno

**Affiliations:** 1 Department of Electrical and Electronic Engineering, Ehime University, Matsuyama, Japan; 2 Pearl Kogyo Co., Ltd., Suminoe, Osaka, Japan; 3 Y’s Corp., Tama, Tokyo, Japan; Universite Toulouse III Paul Sabatier, FRANCE

## Abstract

We have been developing a method of plasma gene transfection that uses microdischarge plasma (MDP) and is highly efficient, minimally invasive, and safe. Using this technique, electrical factors (such as the electrical current and electric field created through processing discharge plasma) and the chemical factors of active species and other substances focusing on radicals are supplied to the cells and then collectively work to introduce nucleic acids in the cell. In this paper, we focus on the electrical factors to identify whether the electric field or electrical current is the major factor acting on the cells. More specifically, we built a spatial distribution model that uses an electrical network to represent the buffer solution and cells separately, as a substitute for the previously reported uniform medium model (based on the finite element method), calculated the voltage and electrical current acting on cells, and examined their intensity. Although equivalent circuit models of single cells are widely used, this study was a novel attempt to build a model wherein adherent cells distributed in two dimensions were represented as a group of equivalent cell circuits and analyzed as an electrical network that included a buffer solution and a 96-well plate. Using this model, we could demonstrate the feasibility of applying equivalent circuit network analysis to calculate electrical factors using fewer components than those required for the finite element method, with regard to electrical processing systems targeting organisms. The results obtained through this equivalent circuit network analysis revealed for the first time that the distribution of voltage and current applied to a cellular membrane matched the spatial distribution of experimentally determined gene transfection efficiency and that the electrical current is the major factor contributing to introduction.

## Introduction

Gene transfection is a technique used for introducing external nucleic acids into cells to express their functionality. These techniques are necessary in a wide variety of fields, including regenerative medicine, drug discovery, and plant breeding [1–5]. Conventional methods of gene transfection can be largely classified into one of three categories: physical methods, chemical methods, and biological methods. In electroporation [6], which is a type of physical gene transfection, electrical pulses are applied to the cell suspension to open small size (less than 20 nm [7]) holes in the cellular membrane and subsequently DNA is physically transferred into the cell. This method requires a temporary but a large variable electric field to create temporary holes on the cellular membrane, thereby resulting in a high rate of cellular death. Genetic damage caused by this large electric field can also result in loss of cell functionality. Another issue with this method is that it requires a certain number of cells and therefore cannot be used for gene transfection with cells attached to a container. In lipofection, which is a common type of chemical gene transfection, genes are enclosed in phospholipid bilayers called liposomes before passing them through the cellular membrane [8]. Phospholipids carrying a positive charge are used to increase affinity with the surfaces of the cellular membrane and improve introduction efficiency. Although genes can be introduced with a high rate of efficiency in cells that are growing, the efficiency of introduction is low for primary cultured cells that have undergone some specialization and have low growth potential. Furthermore, reagents are expensive, and this method is not suited for processing a large number of cells. In a viral vector, which is a type of biological gene transfection, a detoxified virus is used as the means (vector) for delivering genes into cells. The target gene is inserted into the viral genome that then infects cells or tissue, thereby introducing the gene into the target cells [9]. However, because this method involves handling viruses, all operations must be performed at a P2-level recombination experiment facility. It also carries the risk of pathogenic expression and cancerization [10]. Conventional methods of gene transfection have thus presented a range of issues, resulting in the need for a new method of gene transfection that is highly efficient, minimally invasive, and safe.

In 2002, a method of gene transfection using a completely novel concept was discovered wherein cells are exposed to discharge plasma in order to promote gene transfection [11, 12]. This method allows for efficient gene transfection to be performed without inducing cytotoxicity and can even be safely used for gene therapy and other medical uses. In response, researchers began reporting on techniques using various plasma sources and plasma exposure methods to introduce genes and molecules into a range of target cells and substances [13–26]. We discovered the effectiveness of microdischarge plasma (MDP) by utilizing extremely thin electrodes for reliably introducing plasmid DNA with a high molecular weight and demonstrated that this method could provide excellent introduction efficiency as well as a high cell survival rate [27–31]. The electrical factors (such as electric fields and electrical current) and the chemical factors of active species and other substances focusing on radicals produced by plasma act on cells caused endocytosis through the combined effect of both types of factors, resulting in introduction—without introduction occurring even if either factor is activated independently [31]. Focusing on the actions of each factor, Sasaki et al. reported that for small particles such as calcium ions, the activation of TRP channels via short‐lived active species promotes flux into the cell [22]. We employed endocytosis-inhibiting reagents such as MβCD (Sigma-Aldrich) to analyze the introduction mechanism for several sizes of molecules. Although introduction was performed without any impact from endocytosis inhibitors for middle molecules (such as in YOYO-1) and even smaller introduction substances, we observed that endocytosis inhibition had a significant impact on introduction for macromolecules such as plasmid DNA; that is, introduction for large molecules is performed through endocytosis, but another mechanism is utilized for introduction in small and middle molecules. This revealed the need to clarify and differentiate the size of introduction substances when discussing the mechanisms behind plasma molecule introduction phenomena [29–32]. The important issue now is to clarify the effects that the electrical and chemical factors have during introduction with regard to introduction for macromolecules such as plasmid DNA. We herein focus on electrical factors in an attempt to identify the major electrical factor that contributes to gene transfection.

Previously, we had modeled a buffer solution in a 96-well plate as a uniform medium, calculated the electric field distribution in the solution using the finite element method and confirmed that the distribution profiles in the radial direction match for both electric field intensity and introduction efficiency [31]. Providing an electric field with conductivity will help attain electrical current density; thus, the electrical current distribution will have the same profile as the electric field distribution. Therefore, to determine whether electrical current or the electric field is the primary factor in the introduction, we must calculate the electrical current flowing through cells and the voltage applied to cells. This can be achieved using a model that takes the spatial distribution of cells and buffer solution into consideration (rather than a uniform medium) and then discusses not only the distribution profile but also its absolute values.

During our research, we used an electrical network to represent a plasma gene transfection experimental system (comprising electrodes, cells, buffer solution, and a 96-well plate), calculate the electrical current density and electric field, and conduct an analysis. Using an equivalent circuit to create a model (rather than using the finite element method) allows for calculations to be made with few components for both steady analysis and transient analysis. An additional benefit of using an equivalent circuit for analysis is that replacing the system with a simple electrical circuit model makes it easier to initiate phenomena that occur in equivalent parts of the system based on the behavior of the circuit elements. There are existing methods for modeling single cells using electrical circuits in general use [33, 34]. However, to date, there have been no examples of using an electrical network to analyze the spatial distribution of a system with the entire system represented as an equivalent circuit network. In this paper, we calculate the voltage applied and the electrical current flowing through cellular membranes and cytoplasm during electrical network analysis; then, we compare the introduction rate with the spatial distribution of voltage and electrical current and subsequently estimate the major components. We also analyze the absolute values of voltage and electrical current and demonstrate that the major electrical factor behind the mechanism of plasma gene transfection is not the electric field but the electrical current.

## Materials and methods

### Experiment

**Fig 1:**
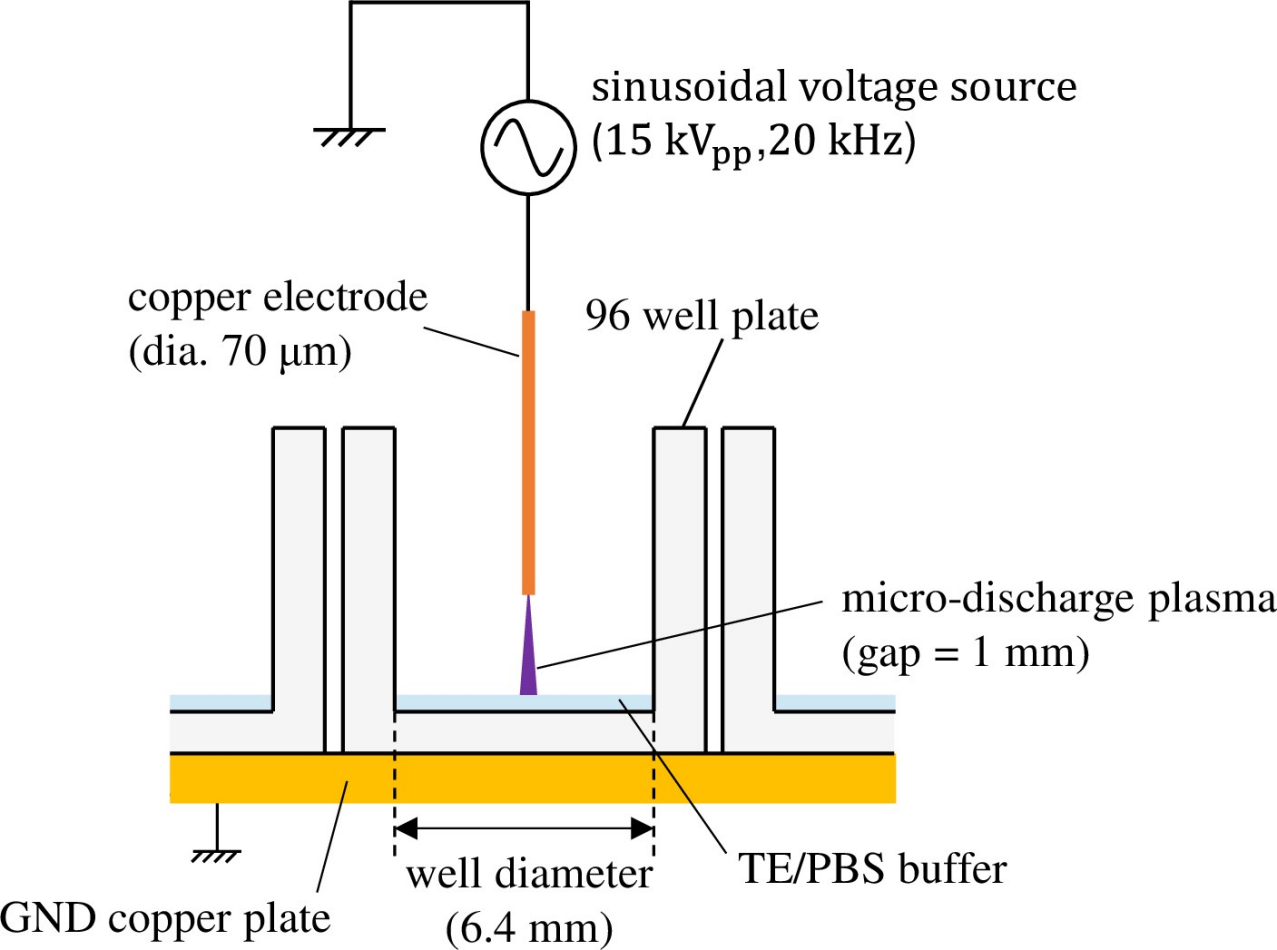
Plasma exposure area of a gene transfection device using microdischarge plasma.

The details of the experimental method used for plasma gene transfection utilizing MDP are provided in Reference 31. Fig 1 shows the structure of the plasma exposure area. The 96-well plate is placed on top of a GND copper plate and a thin high-voltage electrode (diameter: 70 μm) is placed above the center of each well with a vertical gap of 1 mm. Mouse-derived fibroblast L-929 (RCB1422: Riken BRC, Tsukuba, Japan) was cultivated adhering to the bottom surface of each well with 100 μL culture medium for more than 24 h so that it would become confluent. During incubation, the ambient temperature and a CO_2_ concentration were maintained at 37°C and 5% respectively. Before exposing the cells to plasma, the culture medium was extracted; then, 6 μg of plasmids (pAcGFP1-N1: Clontech, Mountain View, CA) were distributed into 6 μL of a TE/PBS buffer solution (adjusted to a conductivity of 0.376 S/m) that was then dripped over the cells. A sinusoidal voltage with an amplitude of 15 kV (peak to peak) and a frequency of 20 kHz was then applied to generate microplasma on the tips of the high-voltage electrodes. Each well was subjected to MDP irradiation for 5 ms. Average input power was (2.9 ± 0.1) W, which means the input energy for 5-ms exposure was (14.7 ± 0.5) mJ.

After a 5-ms exposure to the TE/PBS buffer solution, 100-μL culture medium was added to each well. The sample was left to cultivate for 48 h and then observed with an imaging cytometer (Cytell: GE Healthcare UK, Little Chalfont, UK). Green fluorescent protein was created by introducing plasmids. The number of cells exhibiting green fluorescent light was then counted. For easy evaluation of gene transfection efficiency, the ratio of green pixels to all pixels in a fluorescence image taken with the imaging cytometer was defined as normalized gene transfection efficiency. We employed this protocol because the cultured cells were confluent and uniform. The distribution of plasmid introduction efficiency was then calculated in the radial direction.

### Modeling and calculation

**Fig 2:**
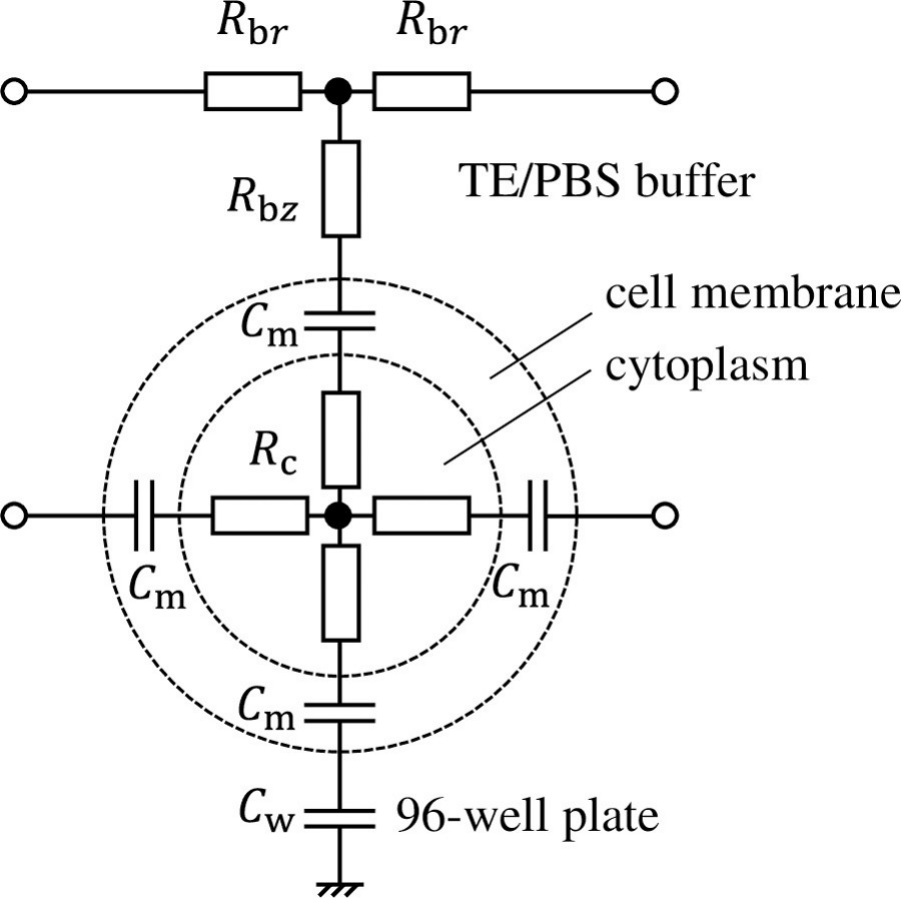
Equivalent circuit comprising a single cell, its surrounding TE/PBS buffer solution, and a 96-well plate.

Fig 2 shows an equivalent circuit comprising a single cell, its surrounding TE/PBS buffer solution, and a 96-well plate. The cell comprises a cellular membrane and cytoplasm, expressed as capacitance *C*_m_ and resistance *R*_c_. Cells are cultured adhered to the bottom surface of the 96-well plate (capacitor *C*_w_) and exposed to plasma after dripping TE/PBS buffer solution (resistor *R*_b*r*_, *R*_b*z*_) on them. The equivalent circuit network that simulates the distribution of voltage and electrical current inside the well is therefore completed when the number of cells in the circuit (shown in Fig 2) covers the bottom surface of the well in a net-like fashion. In reality, some cells stack on other cells so that local multilayers can be present in some area. However, the multilayers are localized in small areas. Thus, the monolayer model is employed in this study.

**Fig 3:**
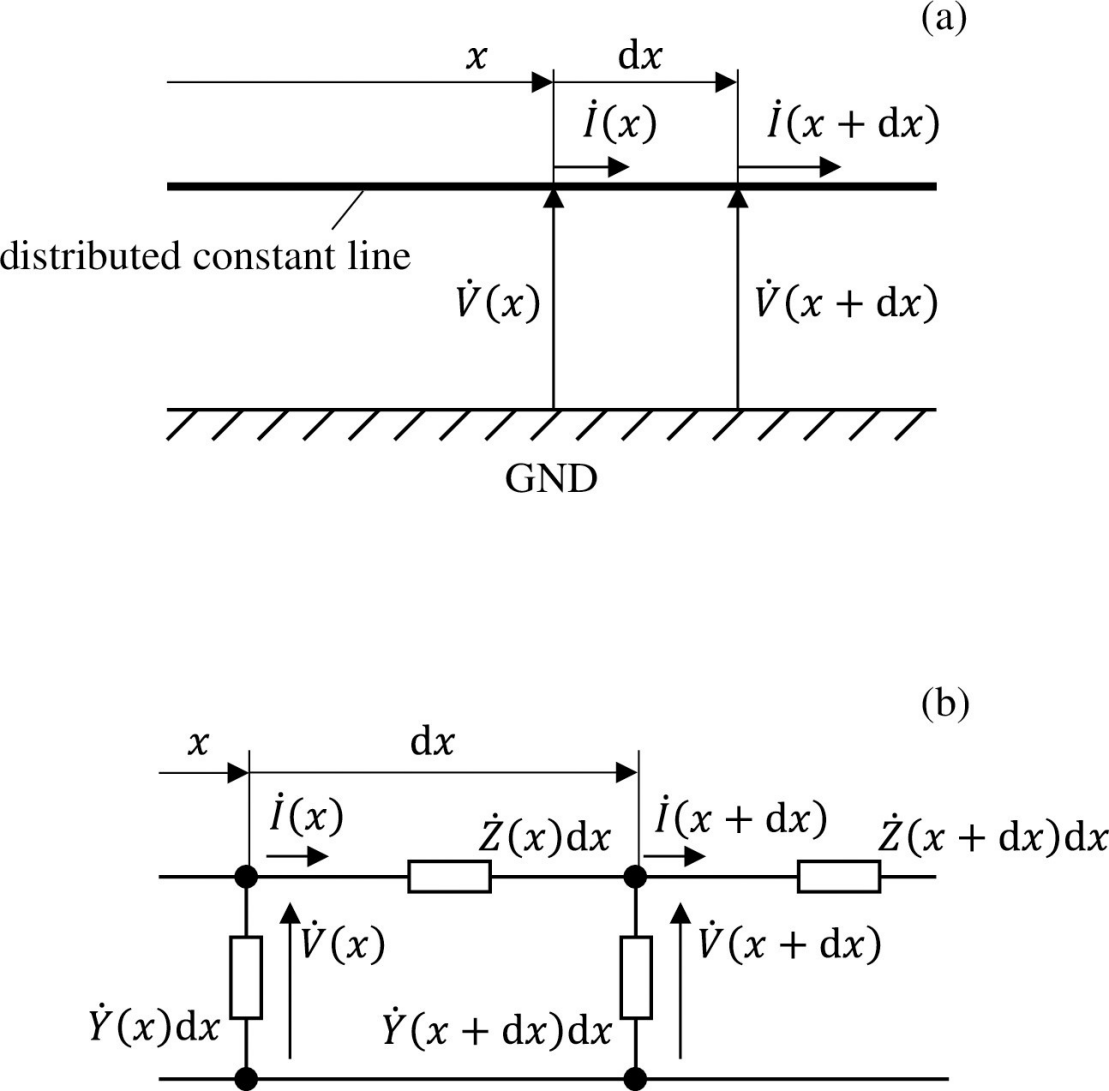
(a) Distribution of voltage/electrical current propagating along a distributed constant line parallel to the GND electrode, and (b) the associated ladder-shaped circuit simulating this.

However, the number of cells in a well would number in the thousands under the cultivation conditions described previously, and it would not be realistic to calculate such a high number. We therefore apply two methods to simplify the equivalent circuit network. First, we assume that the distribution of voltage and electrical current in the well has axial symmetry. This allows us to return to a one-dimensional circuit network model wherein circuit components are connected only in the radial direction. Next, we gather multiple cells into a single circuit element. As shown in Fig 3A, if the wavelengths of the voltage and electrical current signals that transmit a distributed constant line parallel to the GND electrode are sufficiently longer than the spatial size of the circuit, a ladder-shaped circuit can be used for calculation as shown in Fig 3B. Here, $\dot{Z}\left(x\right)$ and $\dot{Y}\left(x\right)$ denote the impedance (serial component) and admittance $\dot{Y}$ (parallel component) per unit transmission length at position *x*. Δ*x* denotes the length of a single component when the transmission line is split into narrow segments; thus, the impedance (serial portion) and admittance (parallel portion) of a single component are expressed as $\dot{Z}\Delta x$ and $\dot{Y}\Delta x$, respectively, while the impedance or admittance of a single component is the impedance or admittance per unit length multiplied by the step size Δ*x*.

**Fig 4:**
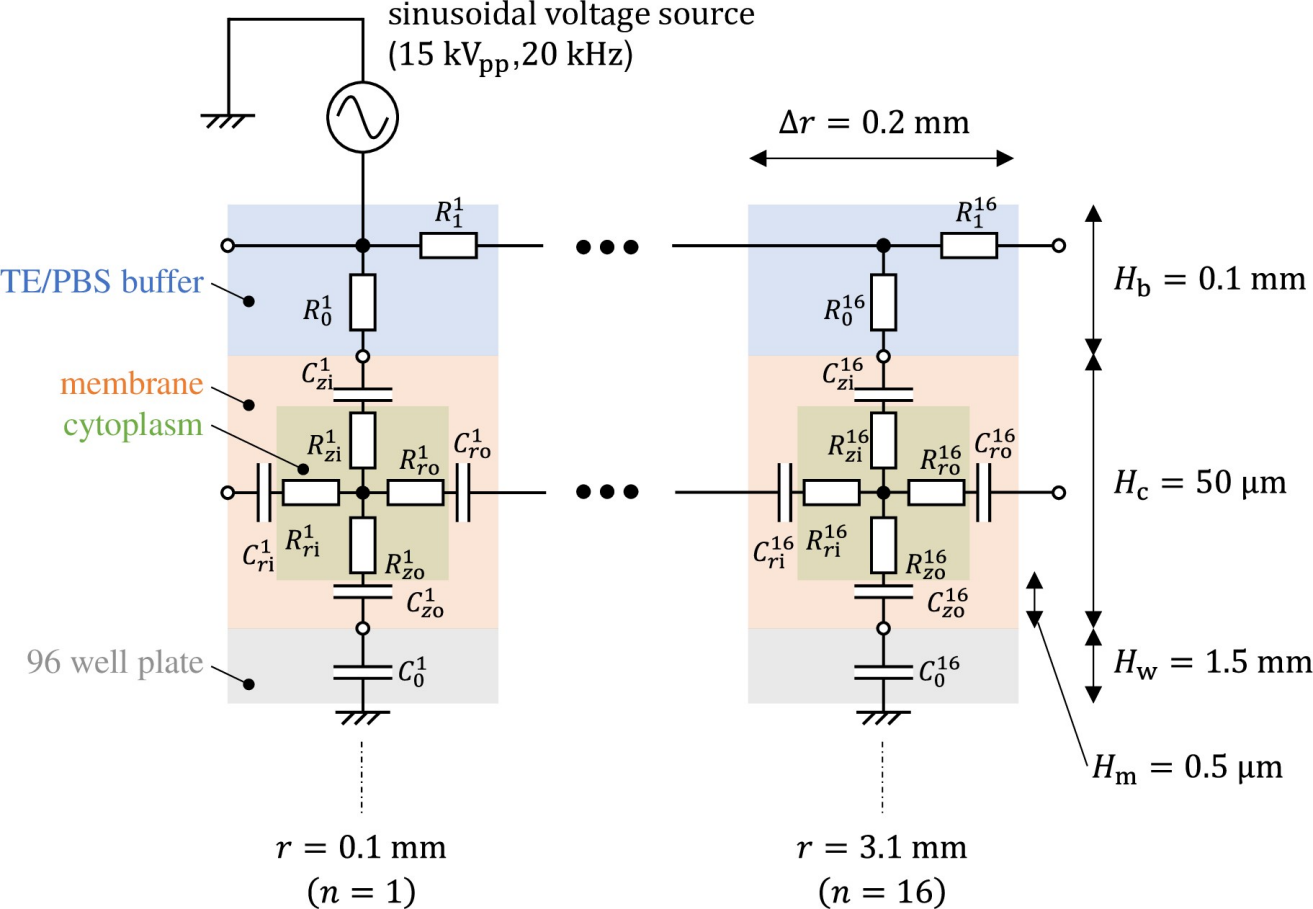
Equivalent circuit network modeling buffer solution, cells, and a 96-well plate for plasma gene transfection.

**Fig 5:**
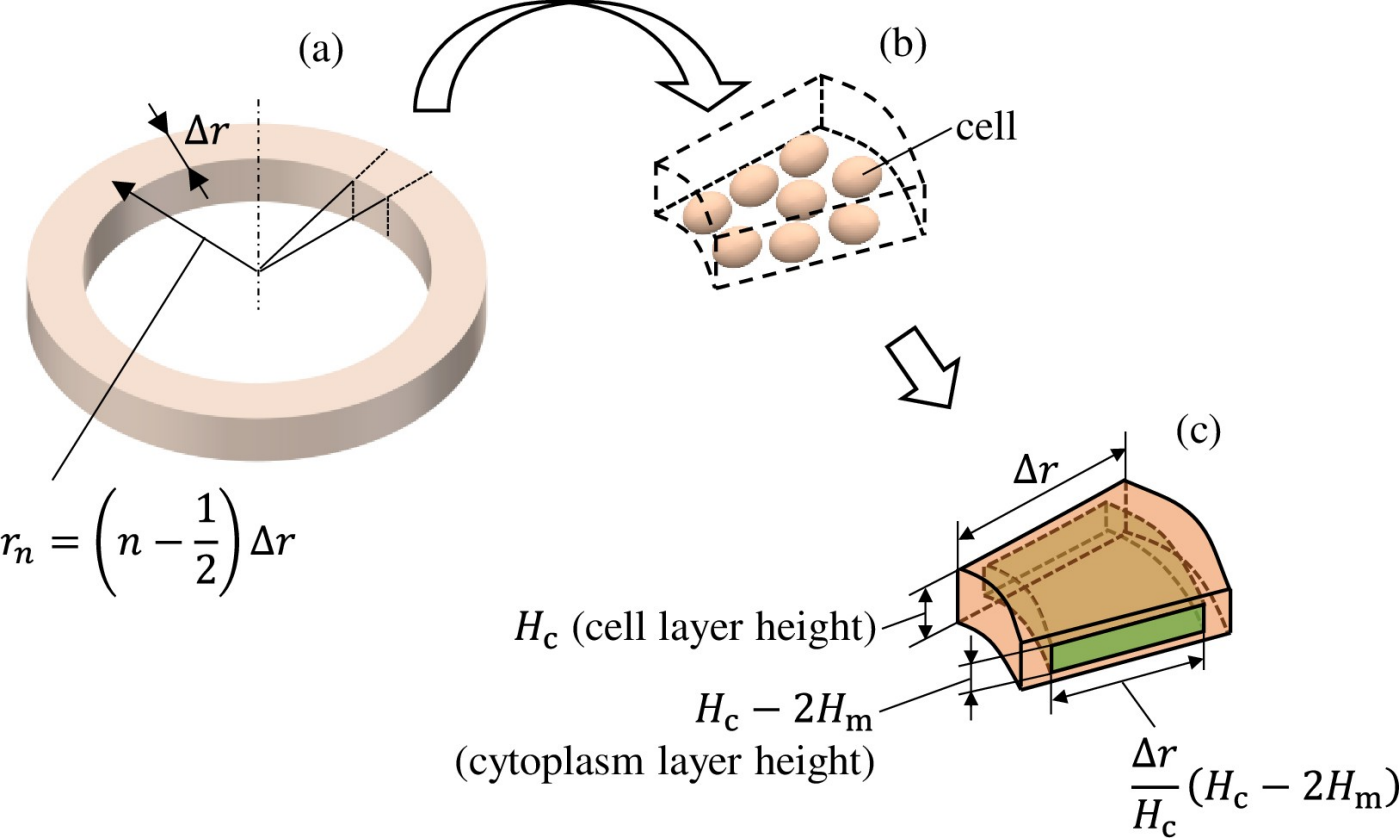
(a) Diagram showing the *n*th component from the cell layer in the equivalent circuit network. (b) Multiple cells are included in a single component. (c) The dimension in the radial direction was determined such that it maintains the ratio of the heights of the cell layer (*H*_c_) and cellular membrane layer (*H*_m_).

The equivalent circuit network obtained through making the above simplifications is shown in Fig 4. It has a three-layer structure comprising a 96-well plate layer, cell layer, and TE/PBS buffer solution layer (in order from the lowest to the highest layer), with the cell layer comprising a cellular membrane and cytoplasm. The height of each layer was set as follows. The 96-well plate layer was set as *H*_w_ = 1.5mm from the actual size; the cell layer was set as *H*_*c*_ = 50 μm from the diameter of the fibroblasts used during the experiment, while the TE/PBS buffer solution layer was set as *H*_*b*_ = 50 mm (the value was determined by dividing the amount of solution by the area of the bottom surface of the well). The cellular membrane layer had a height of *H*_m_ = 0.5 μm, which is 1/100 times the height of the cell layer. There are 16 segments in the radial direction, with each component laid out at a position

$$r_{n}=\left(n-\frac{1}{2}\right)\Delta r\;\left(n=1,\;2,\;\cdots ,\;16\right)$$ (1)

from the center. The length of a single component in the radial direction is Δ*r* = 0.2 mm. Fig 5A is a schematic of the *n*th cell layer. As shown in Fig 5B, there are multiple cells inside this ring distributed in the horizontal direction. Together, these are modeled as a single component in the equivalent circuit shown in Fig 4. The dimension in the radial direction is determined as shown in Fig 5C to maintain the dimensional ratio *H*_c_:*H*_m_ of the cell layer and cellular membrane layer. Therefore, the impedance and admittance of the component shown in Fig 5 are determined by multiplying the impedance and admittance per unit length given by the permittivity of the cellular membrane and the conductivity of the cytoplasm multiplied by the step size Δ*r*.

The values of the element in the equivalent circuit network shown in Fig 4 are therefore determined as follows. The resistance of the TE/PBS layer was

$$R_{0}^{n}=\frac{1}{\sigma_{\text{b}}}\frac{H_{\text{b}}}{2\pi r_{n}\Delta r},\;\left(\text{vertical}\;\text{direction}\right)$$ (2)

$$R_{1}^{n}=\frac{1}{\sigma_{\text{b}}}\frac{\Delta r}{2\pi r_{n}H_{\text{b}}}.\;\left(\text{radial}\;\text{direction}\right)$$ (3)

A conductivity of *σ*_b_ = 0.376 S/m was used according to the conditions of the experiment. The capacitance of the cellular membrane layer is

$$C_{z\text{i}}^{n}=C_{z\text{o}}^{n}=\varepsilon_{\text{m}}\frac{2\pi r_{n}\Delta r}{H_{\text{m}}},\;\left(\text{vertical}\;\text{direction}\right)$$ (4)

$$C_{r\text{i}}^{n}=C_{r\text{o}}^{n}=\varepsilon_{\text{m}}\frac{2\pi r_{n}H_{\text{c}}}{\Delta r/H_{\text{c}}\cdot H_{\text{m}}}.\;\left(\text{radial}\;\text{direction}\right)$$ (5)

The resistance of the cytoplasm layer is

$$R_{z\text{i}}^{n}=R_{z\text{o}}^{n}=\frac{1}{\sigma_{\text{c}}}\;\frac{\left(H_{\text{c}}-2H_{\text{m}}\right)/2}{2\pi r_{n}\cdot \Delta r/H_{\text{c}}\cdot \left(H_{\text{c}}-2H_{\text{m}}\right)},\;\left(\text{vertical}\;\text{direction}\right)$$ (6)

$$R_{r\text{i}}^{n}=R_{r\text{o}}^{n}=\frac{1}{\sigma_{\text{c}}}\;\frac{\Delta r/H_{\text{c}}\cdot \left(H_{\text{c}}-2H_{\text{m}}\right)/2}{2\pi r_{n}\;\left(H_{\text{c}}-2H_{\text{m}}\right)}.\;\left(\text{radial}\;\text{direction}\right)$$ (7)

The cellular membrane permittivity *ε*_m_ and cytoplasm conductivity *σ*_c_ were each set to *ε*_m_ = 30*ε*_0_ and *σ*_c_ = 1 S/m, respectively, with reference to the values in Reference 34. *ε*_0_ is the permittivity in vacuum. Based on the polystyrene capacitance *ε*_w_ = 2.4*ε*_0_, the capacitance of the 96-well plate layer was set to

$$C_{0}^{n}=\varepsilon_{\text{w}}\frac{2\pi r_{n}\Delta r}{H_{\text{w}}}.$$ (8)

The plasma approximates a conductor of with zero resistance, and a sinusoidal voltage source with an amplitude of 15 kV (peak to peak) and frequency of 20 kHz was connected directly to the *n* = 1st component in the TE/PBS buffer solution layer.

Using this model, we calculated the voltage and electrical current of each element using LTspice and then calculated the distribution in the radial direction of the electric field intensity and the electrical current density divided by the length or cross-sectional area of the component corresponding to each element.

## Results

**Fig 6:**
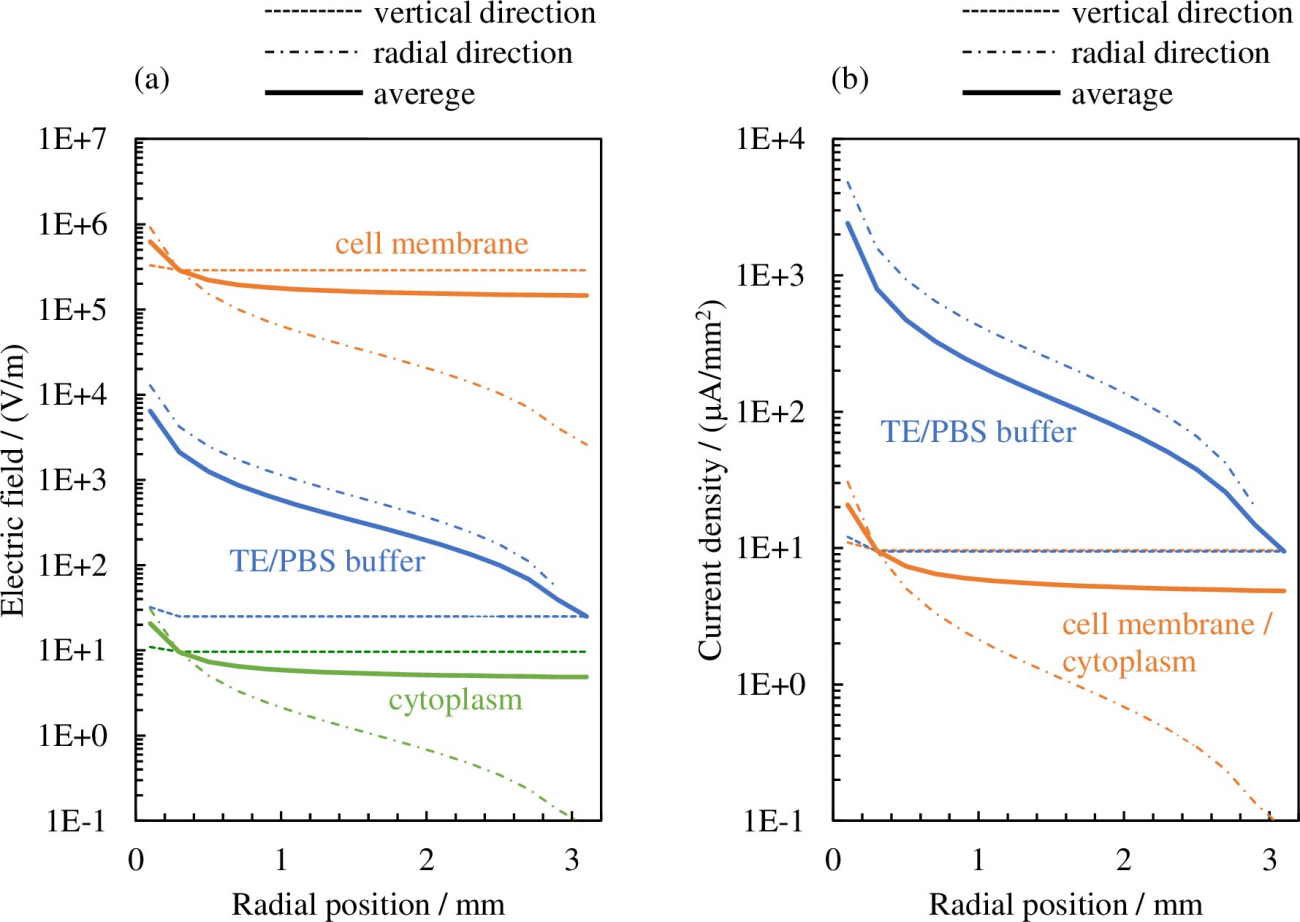
Distribution in the radial direction of the effective values of (a) the electric field and (b) electrical current density.

Fig 6 shows the results of calculating the distribution in the radial direction of the effective values of the electric field and electrical current density. The dotted lines and dashed–dotted lines indicate the values calculated from the elements connected in the vertical direction ($R_{0}^{n}$, $C_{z\text{i}}^{n}$, $C_{z\text{o}}^{n}$, $R_{z\text{i}}^{n}$, $R_{z\text{o}}^{n}$) and the radial direction ($R_{1}^{n}$, $C_{r\text{i}}^{n}$, $C_{r\text{o}}^{n}$, $R_{r\text{i}}^{n}$, $R_{r\text{o}}^{n}$), respectively, in Fig 4. The solid lines indicate the average values of the two portions. If we take the electrical current density of the cellular membrane as an example, these values have the following meaning in a physical sense.

If the effective value of the electrical current density vector on the cellular membrane surface S is ***J***_c_, the value of the electrical current density passing through the cellular membrane and averaged spatially can be determined as follows:

$$J_{\text{c}}=\frac{1}{S}\underset{\text{S}}{\oint }\left|J_{\text{c}}\cdot \text{d}S\right|.$$ (9)

However, *S* is the surface area of the cellular membrane, and d***S*** is the surface component vector on the cellular membrane surface S. If we assume the shape of the cell to be a sphere of diameter *H*_c_, Eq 9 can be written as follows.

$$J_{\text{c}}=\frac{2S^{\prime }J_{\text{c}r}+2S^{\prime }J_{\text{c}z}}{S}=\frac{J_{\text{c}r}+J_{\text{c}z}}{2}.$$ (10)

*J*_*cr*_ and *J*_cz_ denote the *r* and *z* portions of ***J***_c_, respectively, and the surface area is therefore $S=\pi H_{\text{c}}^{2}$. $S^{\prime }=\pi H_{\text{c}}^{2}/4$ is the surface of the planar projections of the cell (assumed to be a sphere with the diameter *H*_c_) in the *r* and *z* directions. Therefore, the electrical current density passing through the cellular membrane is expressed as the average value of both the *r* and *z* portions. Similarly, the average values of both the *r* and *z* portions are denoted with solid lines for the electric field outside the cellular membrane.

If we next consider the admittance in the unit length and unit cross-sectional area (hereafter simply referred to as “admittance”), the TE/PBS buffer solution (*σ*_b_ = 0.376 S/m) is significantly larger than the cellular membrane (*ωε*_m_ = 33.4 μS/m), and a considerable portion of the electrical current supplied by the plasma will flow into the TE/PBS buffer solution with some portion branching in the vertical direction and flowing into the cell (Fig 6B). The amount of electrical current density branched in the vertical direction will be approximately the same regardless of the distance from the center, as shown below. If we next consider the impedance of elements along the vertical route in Fig 4, the impedance of the 96-well plate (1/(*ωε*_w_) = 374 kΩ m) is significantly larger in comparison; consequently, the majority of the voltage is applied by dividing it to the 96-well plate. The distribution of the vertical components of the electrical current density and electric field in the radial direction will therefore be approximately uniform.

**Fig 7:**
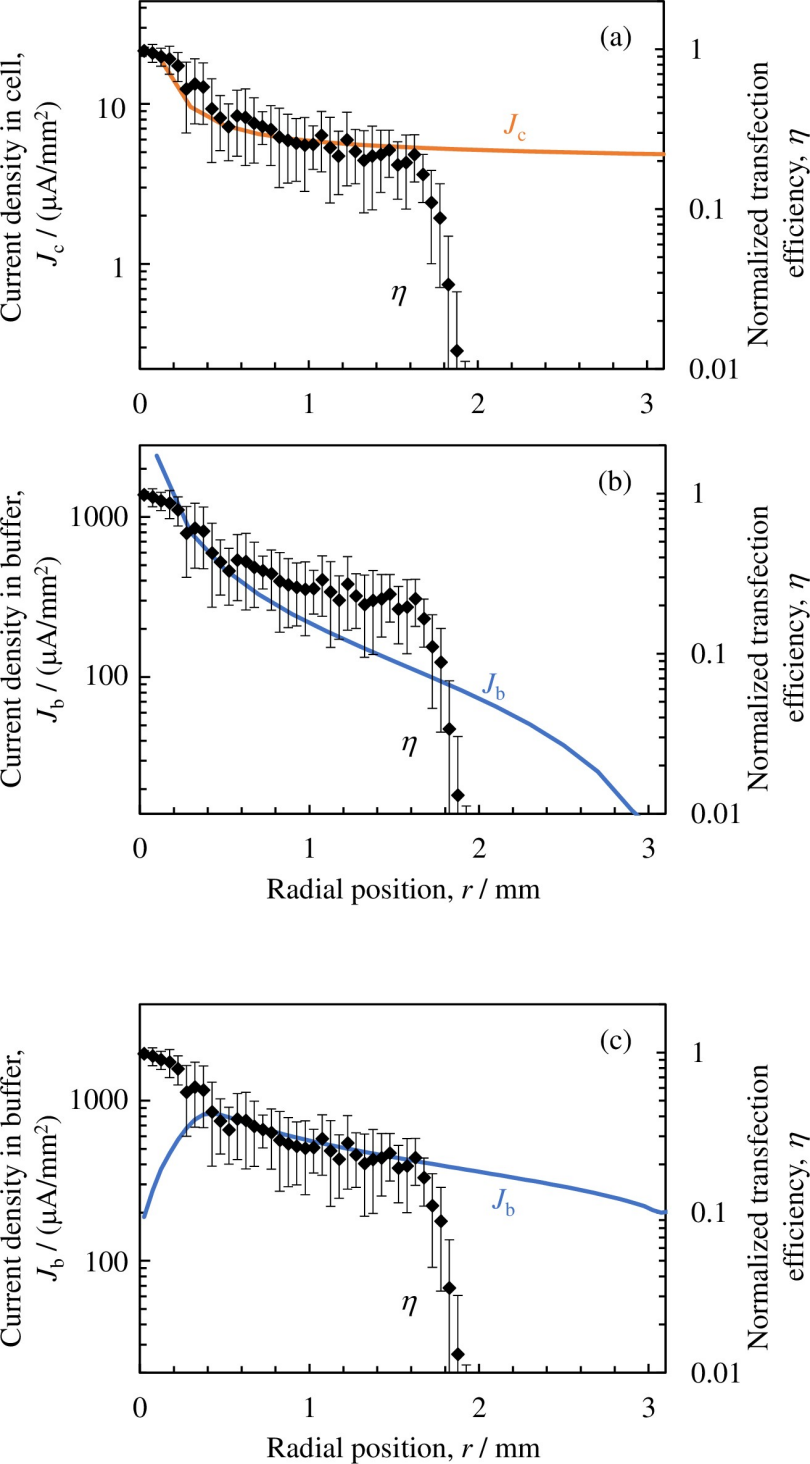
Distribution in the radial direction of the effective values of the electrical current density for (a) the cell (*J*_c_) and (b) the TE/PBS buffer solution (*J*_b_), and normalized gene transfection efficiency (*η*) [31]. The electrical current density in the TE/PBS buffer solution derived from finite element method [31] is also shown in Fig 7C.

To verify that electrical factors contribute during the gene transfection process, we plotted the distribution of the normalized gene transfection efficiency in the radial direction for the electrical current density (the average values in both the vertical and radial directions) overlapped on the graphs shown in Fig 7. The electrical current density in cell (*J*_c_) and in buffer solution (*J*_b_) are shown in Fig 7A and 7B, respectively. For comparison, the electrical current density in buffer solution calculated by finite element method is also shown in Fig 7C; that was derived from the electric field shown in Ref. 31 multiplying conductivity of the buffer solution (*σ*_b_ = 0.376 S/m). Although the vertical axis spans for the current density in Fig 7A–7C are same (200 times), each vertical axis range is adjusted so that the current density and the transfection efficiency overlap in wide range; they overlap in range of *r* < 1.6 mm for (a), 0.2 mm < *r* < 0.5 mm for (b), and 0.4 < *r* < 1.6 mm for (c). The normalized transfection efficiencies (*η*) in Fig 7A–7C are identical and were taken from Ref. 31. Note that the electrical current density *J* and electric field *E* are expressed as *J* = *σE* (TE/PBS buffer solution or cytoplasm) or *J* = *ωεE* (cellular membrane); thus, the distribution profiles in the radial direction match for both the electrical current density and the electric field. Fig 7A and 7B show that although the distribution profiles of the electrical current density flowing into (or the electric field applied to) the cell match the introduction rate distribution profile within the range of *r* < 1.6 mm, the density of the electrical current flowing into the TE/PBS buffer solution shows a different distribution profile than that of the introduction rate. In the range of *r* < 0.4 mm, the current density derived from finite element method (Fig 7C) shows different behavior compared with that derived from the circuit network model (Fig 7A and 7B). This is due to the plasma attaching to this region, which is considered as a conductor (*σ* = 1 S/m). The discharge current is uniformly supplied to the top surface of the buffer solution in this region, whereas in the circuit network model, it is supplied to the first circuit unit (*n* = 1 in Fig 4), which covers the region of *r* < 0.2 mm.

## Discussion

Within the range of *r* < 1.6 mm, the distribution profiles in the radial direction for the density of the electrical current flowing to the cell or the electric field applied to the cell are the same as the distribution profile in the radial direction for the introduction rate. The results therefore suggest that the electrical factors on the cell contribute toward gene transfection. The electrical current densities in the TE/PBS buffer derived from two models show different distribution (Fig 7B and 7C). Furthermore, the differing distribution profiles for the electrical current density for the cell and TE/PBS buffer solution clearly demonstrate that conducting an analysis using a spatial distribution model representing the TE/PBS buffer solution separated from the cell, instead of using the uniform medium model demonstrated in Reference 31, is important.

**Fig 8:**
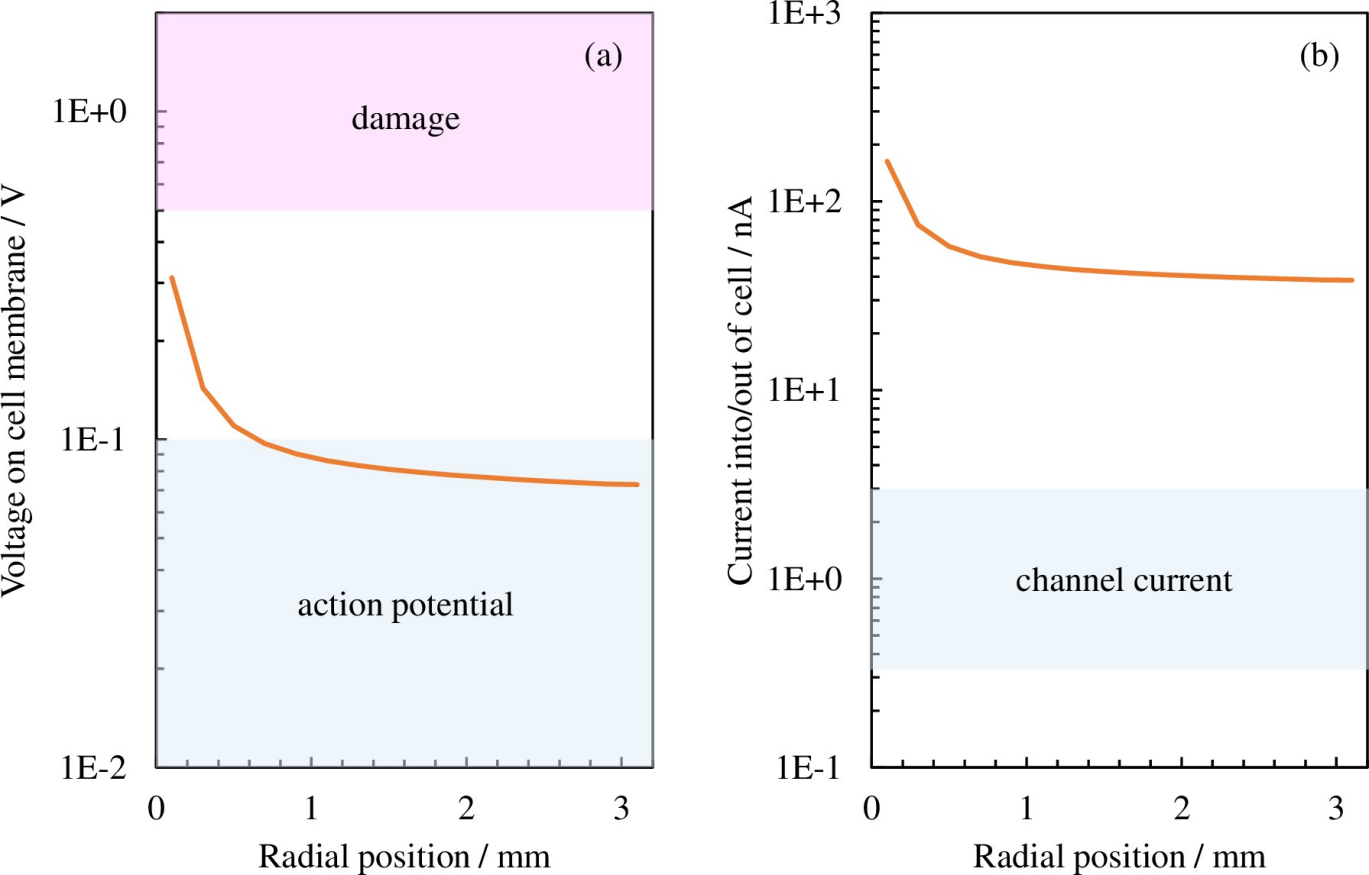
Distribution of effective values in the radial direction for (a) the voltage applied to the cellular membrane and (b) the electrical current flowing to the cell. The blue shaded regions in (a) and (b) indicate the action potential (approximately 0.1 V or lower) induced through normal cell activity and the electrical current value (of the order of 1 nA) caused by membrane transport through the ion channel, respectively. The red shaded region indicates the voltage (approximately 0.5 V or higher) wherein the cellular membrane would be damaged.

Plasmids are introduced in the cell through the cellular membrane; therefore, we next compare the voltage and electrical current values during cellular membrane transport, which are generally known, with the values calculated using this model. An electrical current of the order of approximately 1 nA flows during membrane transport for basic ions such as potassium and sodium that have passed through the ion channel, thereby creating a membrane potential of around 0.01–0.1 V. Applying a voltage exceeding approximately 0.5–1 V to the cellular membrane will cause irreversible damage to it and bring about cellular death [35–43]. We next take the electric field of the cellular membrane shown in Fig 6 and convert it into voltage by multiplying the cellular membrane width by *H*_m_ (shown in Fig 8A); next, we convert the surface area $\pi H_{\text{c}}^{2}$ assuming a spherical cell into electrical current by multiplying it by the electrical current density (shown in Fig 8B). The blue shaded regions in the figure indicate the action potential (approximately 0.1 V or lower) induced through normal cell activity and the electrical current value (of the order of 1 nA) caused by membrane transport through the ion channel. The red shaded region indicates the voltage (approximately 0.5 V or higher) at which the cellular membrane would be damaged. If we exclude the area around the center, a voltage less than that which would cause membrane damage (0.5–1 V) is applied to most regions, with a voltage around the same order as that during ion membrane transport applied to the cellular membrane. We now consider electrical current. The plasma supplies an electrical current that is two digits larger than that during ion membrane transport. This shows that electrical current density is the major acting factor among the electrical factors applied during the plasma gene transfection process.

However, the distribution profiles for the introduction rate and electrical current density do not match within the range of the area around the well (*r* > 1.6 mm). Both electrical and chemical factors are required during the plasma gene transfection process, and it is through the combined effect of these that introduction occurs [31]. In contrast with how the electrical factors (electrical current density or the electric field) are consecutively distributed up to the edge of the well, the distribution of the introduction rate decreases drastically around *r* = 1.6 mm and drops to nearly zero outside that. This suggests that, although there are sufficient chemical factors in the region where *r* < 1.6 mm and the introduction rate is determined here via the electrical current, the active species required to express the combined effect are not transported to the region where *r* > 1.6 mm. Our next challenge will be to create a model that includes the transportation of active species.

## Conclusion

In this paper, we analyzed the electrical factors that work together with chemical factors in the plasma gene transfection method using MDP. We built a spatial distribution model that uses an electrical network to represent the buffer solution and cells separately, as a substitute for the uniform medium model (based on the finite element method) demonstrated previously; subsequently, we calculated the voltage and electrical current acting on the cells. Using this model, we could calculate electrical factors using fewer components than those required for the finite element method. We also revealed that the distribution of voltage and current applied to a cellular membrane matched the spatial distribution of the experimentally determined gene transfection efficiency and that electrical current is the major factor contributing to the introduction of nucleic acids in the cells.

## Supporting information

S1 Table Data for Fig 6. (XLSX)

S2 Table Data for Fig 7. (XLSX)

S3 Table Data for Fig 8. (XLSX)
